# Transcriptome sequencing reveals altered ciliogenesis under hypoxia in nasal epithelial cells from chronic rhinosinusitis with nasal polyps

**DOI:** 10.1002/clt2.12168

**Published:** 2022-06-08

**Authors:** Jian Jiao, Puqi Hu, Mengyan Zhuang, Ying Li, Chao Cai, Xiangdong Wang, Luo Zhang

**Affiliations:** ^1^ Department of Otolaryngology Head and Neck Surgery Beijing TongRen Hospital Capital Medical University Beijing China; ^2^ Beijing Key Laboratory of Nasal Diseases Beijing Institute of Otolaryngology Beijing China; ^3^ Department of Otolaryngology Beijing You'an Hospital Capital Medical University Beijing China

**Keywords:** ciliogenesis, human nasal epithelial cells (HNECs), hypoxia, transcriptome sequencing

## Abstract

**Background:**

Hypoxia is considered a key factor in the pathogenesis of chronic rhinosinusitis with nasal polyps (CRSwNP). However, the specific mechanism driving polypogenesis under hypoxic conditions is unclear. This study aimed to explore hypoxia‐induced alterations in the transcriptome of human nasal epithelial cells (HNECs) in vitro.

**Methods:**

HNECs derived from the tissue of patients with CRSwNP were established as air–liquid interface (ALI) cultures. Confluent cultures were kept submerged or treated with cobalt chloride (CoCl2) to induce hypoxia. Transcriptome analysis was used to identify key mRNAs involved in this process. Real‐time PCR (RT–PCR), Western blotting, and immunofluorescence were used to observe the effects of hypoxia on ciliogenesis.

**Results:**

Numerous genes, biological processes and pathways were altered under submerged culture conditions or after CoCl2 treatment. Analysis of the results under both hypoxic conditions revealed that the transcriptional program responsible for ciliogenesis was significantly impaired. Downregulation of cilia‐related genes and inhibition of ciliated cell differentiation under hypoxia were confirmed by RT–PCR, Western blot and immunofluorescence analyses.

**Conclusion:**

Hypoxia impairs ciliogenesis and ciliary function in HNECs, which might play a role in the pathogenesis of CRSwNP.

## 1 INTRODUCTION

Chronic rhinosinusitis (CRS) is a multifunctional inflammatory disease of the nasal cavity and sinus mucosa lasting more than 12 weeks, and it imposes a heavy burden not only on individual patients but also on the economy and society.^1^, ^2^ CRS with nasal polyps (CRSwNP) accounts for approximately 20% of CRS cases and has more severe clinical symptoms and a higher recurrence rate.^2^, ^3^ However, the pathogenesis of CRSwNP is still largely unclear.

The upper respiratory epithelium constitutes the first line of defense against inhaled irritants, pathogens and allergens. Normal sinonasal epithelium is a pseudostratified columnar ciliated epithelium comprising three main cell types: multiciliated cells, mucus‐secreting goblet cells and basal cells. In addition to serving as a physical barrier via apical junctions and mucociliary clearance, the epithelium also forms a chemical and immunological barrier to protect the airway against external attacks.^4^ Disrupted epithelial function has been shown to contribute to the pathogenesis of CRSwNP^4^, ^5^, ^6^, ^7^; more importantly, a recent study by Callejas‐Díaz et al. demonstrated that the genetic transcriptional program that is responsible for ciliogenesis and cilia function was significantly impaired in CRSwNP epithelium^8^; however, the mechanisms underlying such abnormalities are not known. Therefore, a better understanding of the mechanism of epithelial dysfunction might facilitate the identification of new epithelial‐targeted therapies.

Hypoxia is defined as decreased availability of oxygen in tissues, and a hypoxic state in the airway epithelium has been reported to be associated with several chronic airway diseases, such as allergic rhinitis, asthma, and chronic obstructive pulmonary disease.^9^ Moreover, hypoxia is considered to play an important role in the pathogenesis of CRS, and oxygen levels were found to be substantially decreased in the sinus cavities of patients with CRS.^10^ The expression of hypoxia‐inducible factor (HIF)‐1α, an essential factor for oxygen homeostasis and a well‐known hypoxia marker, has been reported to be upregulated in the sinus mucosal epithelium in patients with CRSwNP.^11^ Recent study by Zhong et al.^12^ demonstrated that HIF‐1α induces the expression and activation of pyrin domain containing 3 (NLRP3) inflammasome by M1 macrophages in noneosinophilic CRSwNP, contributing to CRSwNP pathogenesis. Additional studies have reported that hypoxia can induce mucus hyperproduction, epithelial barrier disruption and tissue remodeling in the upper airway epithelium.^9^ Despite these findings, the effects of hypoxia on human nasal epithelial cells (HNECs) and the potential underlying regulatory mechanism are incompletely elucidated.

The aim of the present study was to establish an in vitro model of hypoxia in HNECs and to investigate the alterations in epithelial cell function under hypoxia by transcriptome analysis to identify the key genes, biological processes (BPs) and pathways regulated during this process.

## 2 MATERIALS AND METHODS

### 2.1 Subjects

Ten subjects with CRSwNP were recruited for this study. Patients with CRSwNP were diagnosed according to the European Position Paper on Rhinosinusitis and Nasal Polyps 2020 (EPOS 2020) guidelines.^1^ The diagnosis of allergic rhinitis was evaluated based on Allergic Rhinitis and its Impact on Asthma (ARIA) 2008 update.^13^ The diagnosis of comorbid asthma was made according to the Global Initiative for Asthma 2019 guidelines. Subjects with severe uncontrolled systemic diseases, immunodeficiency, fungal sinusitis, and aspirin‐exacerbated respiratory disease (AERD) were excluded. None of the patients had been treated with oral/nasal corticosteroids or antibiotics during the 4 weeks before surgery. Additionally, none of the patients had received biologics during the 6 months before surgery. The demographics and clinical characteristics of these patients are shown in Table S1. Nasal polyp tissues were isolated from patients with CRSwNP during surgery. The Ethics Committee of Beijing Tongren Hospital (TRECKY2019‐050) approved this study, and all patients signed informed consent forms before enrollment.

### 2.2 Cell culture and hypoxia model establishment

Primary HNECs were cultured as previously described.^14^ In brief, polyp tissues obtained during surgery were dissociated in 0.1% protease type XIV solution (Sigma–Aldrich, St Louis, MO, USA) overnight at 4°C. Purified epithelial cells (1 × 10^5^ cells per well) were seeded onto human placental collagen (Sigma–Aldrich, St Louis, MO, USA)‐precoated, 6.5 mm‐diameter, 0.4 µm‐pore size polyester membrane Transwell inserts (Costar, Corning, NY, USA) in bronchial epithelial growth medium (BEGM):DMEM (1:1). At confluence (5–7 days), air‐liquid interface (ALI) culture was established by removing the medium on the apical side and feeding cells with BEGM:DMEM (1:1) containing 50 nM all‐trans retinoic acid solely on the basolateral side. The ALI culture medium in the basolateral compartment was replaced every other day.

The establishment of the hypoxia model was initiated 7 days after the establishment of the ALI culture. We used submerged culture and CoCl2 treatment to induce cellular hypoxia, as described in other publications.^15^, ^16^, ^17^ Specifically, cell cultures from 5 CRSwNP patients were used to establish the submerged culture hypoxia model by exposing cells to 100 µL of ALI medium (added to the apical compartment for 4 h daily for 7 days), whereas the control cells were maintained at a ALI without treatment (ALI control, ALI). Cell cultures from another 5 CRSwNP patients were used to establish the cobalt chloride (CoCl2) mimetic hypoxia model by treating cells with 100 µM CoCl2 (added to the ALI culture medium in the basolateral compartment; Sigma–Aldrich, St Louis, MO, USA) for 7 days. The control cells were untreated by the addition of only ALI culture medium to the basolateral compartment (Untreated control, Untreated). After 7 days of hypoxia treatment, cell cultures were harvested for downstream analysis. The timeline scheme of this study design is shown in Figure S1.

### 2.3 RNA sequencing (RNA‐seq) and data analysis

For RNA‐seq, total RNA extraction, library construction, sequencing and data analysis were carried out by Novogene (Beijing, China). RNA‐seq was performed on the Illumina NovaSeq platform. Raw data were filtered to remove reads containing adapters, reads containing poly(N) sequences and low‐quality reads. Reference genome and gene model annotation files were downloaded directly from the genome website. HISAT2 v2.0.5 was used to build an index of the reference genome and align the clean paired‐end reads to the reference genome. The numbers of reads mapped to each gene were determined using featureCounts v1.5.0‐p3, and the fragments per kilobase of transcript per million mapped reads (FPKM) value of each gene was then calculated according to the length of the gene and the number of reads mapped to the gene. Differential expression analysis was performed using the DESeq2 R package (1.20.0). Genes with a p value of less than 0.05 and a log2 fold change of greater than 1 or less than −1 were considered differentially expressed. Differentially expressed genes were further subjected to Gene Ontology (GO) BP and Kyoto Encyclopedia of Genes and Genomes (KEGG) pathway enrichment analyses with the clusterProfiler R package. GO BPs and KEGG pathways with a *p* value < 0.05 were considered significantly enriched.

### 2.4 Real‐time polymerase chain reaction (RT–PCR)

After incubation, HNECs were lysed in RLT buffer, and total RNA was extracted using an RNeasy Kit (Qiagen, Hilden, Germany). Complementary DNA (cDNA) was synthesized using PrimeScriptTM RT Master Mix (TaKaRa Biotechnology). RT–PCR was performed using a SYBR Green assay (TaKaRa Biotechnology) according to the manufacturer's instructions. Glyceraldehyde 3‐phosphate dehydrogenase (GAPDH) was used as the endogenous reference for mRNAs. Relative expression was calculated using the comparative cycle threshold method. The sequences of the primers used to amplify the target genes are presented in Table S2.

### 2.5 Western blot analysis

After hypoxia treatment, HNECs were harvested by using RIPA lysis buffer containing 1% protease inhibitor for 30 min. The protein concentration was measured with a BCA kit (Beyotime, Shanghai, China). Then, cell lysates containing equal amounts of protein (20 µg) were separated by SDS–PAGE and transferred to PVDF membranes. The membranes were blocked with 5% skim milk and incubated overnight at 4°C with anti‐HIF‐1α (1:1000, BD Biosciences), anti‐acetylated α‐tubulin (1:500, Sigma–Aldrich), and anti‐GAPDH (1:10,000, Abcam) antibodies. Then, the membranes were incubated with horseradish peroxidase‐conjugated secondary antibodies for 60 min at room temperature and exposed to enhanced chemiluminescence (ECL) solution, and chemiluminescence was detected in a ChemiDocTM MP Imaging System (Bio–Rad, UK). Band density was analyzed by using the Image Lab software Version 6.0.0 (Bio‐Rad, UK).

### 2.6 Immunofluorescence staining

HNECs were sequentially fixed with a 1:1 acetone:methanol mixture for 10 min, permeabilized with 0.3% Triton X‐100, blocked with 5% skim milk, and incubated overnight at 4°C with an anti‐β‐tubulin IV antibody (1:500, Sigma–Aldrich). The secondary antibody was labeled with fluorescein isothiocyanate (FITC) (1:500, Invitrogen). Then, the specimens were counterstained with 4′,6‐diamidino‐2‐phenylindole (DAPI) for nuclear staining and examined under a confocal microscope (Model IX81; Olympus, Tokyo, Japan). The number of β‐tubulin IV‐positive cells in 10 random fields of vision per culture was counted and expressed as the percentage of the total cells.

### 2.7 Statistical analysis

GraphPad Prism Version 5.0 software (GraphPad Software, La Jolla, CA, USA) was used for the data analysis. All of the data are expressed as means ± SEM, unless otherwise noted. The measurement data were normally distributed and were statistically analyzed by using a paired *t* test between the control and treatment groups. A two‐tail value of p < 0.05 was considered to be statistically significant.

## 3 RESULTS

### 3.1 Differential mRNA expression under hypoxic conditions

**FIGURE 1:**
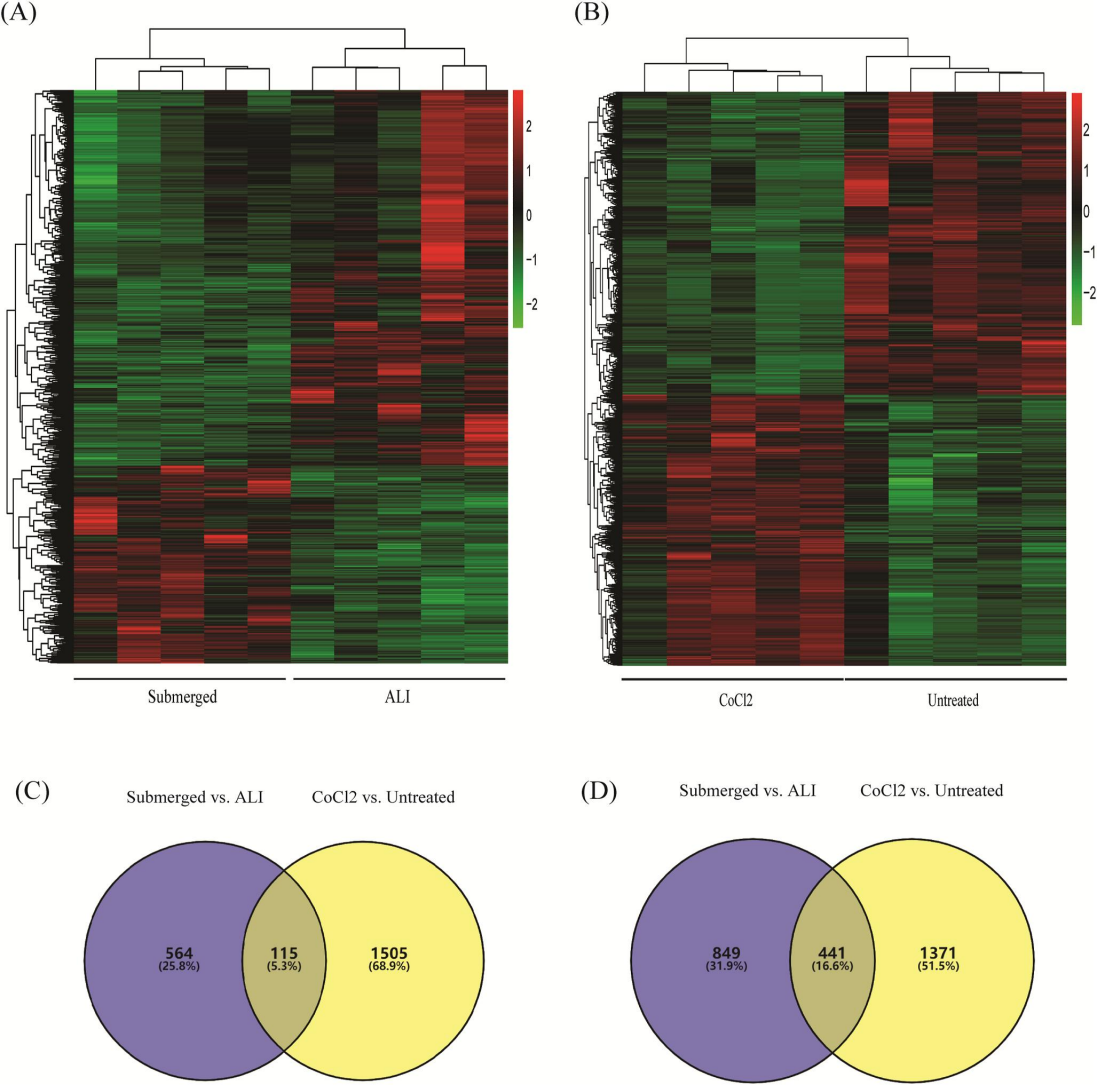
DE‐mRNAs under hypoxia. Heatmaps showing the expression of DE‐mRNAs in HNECs after 7 days of submerged culture (Submerged) compared to the ALI control (ALI) HNECs (*n* = 5) (A), or the expression of DE‐mRNAs in HNECs after 7 days of 100 µM CoCl2 treatment (CoCl2) compared to untreated control (Untreated) HNECs (*n* = 5) (B). Red indicates a high expression level, and green indicates a low expression level. Venn diagrams showing upregulated (C) and downregulated (D) DE‐mRNAs in HNECs in the Submerged versus ALI groups and the CoCl2 versus Untreated groups. The numbers of DE‐mRNAs are indicated in the corresponding areas

After 7 days of submerged culture or CoCl2‐induced hypoxia, RNA‐seq was performed to generate mRNA expression profiles. The analysis of differentially expressed mRNAs (DE‐mRNAs) identified 1969 DE‐mRNAs (679 upregulated and 1290 downregulated) between HNECs in the submerged culture (Submerged) and HNECs in the ALI control (ALI), as well as 3432 DE‐mRNAs (1620 upregulated and 1812 downregulated) between 100 µM CoCl2‐treated HNECs (CoCl2) and untreated control HNECs (Untreated) (Figure 1). Furthermore, 556 DE‐mRNAs, namely, 115 upregulated and 441 downregulated DE‐mRNAs, were shared between the two hypoxic conditions (Figure 1C,D).

### 3.2 Functional enrichment analysis of DE‐mRNAs

**FIGURE 2:**
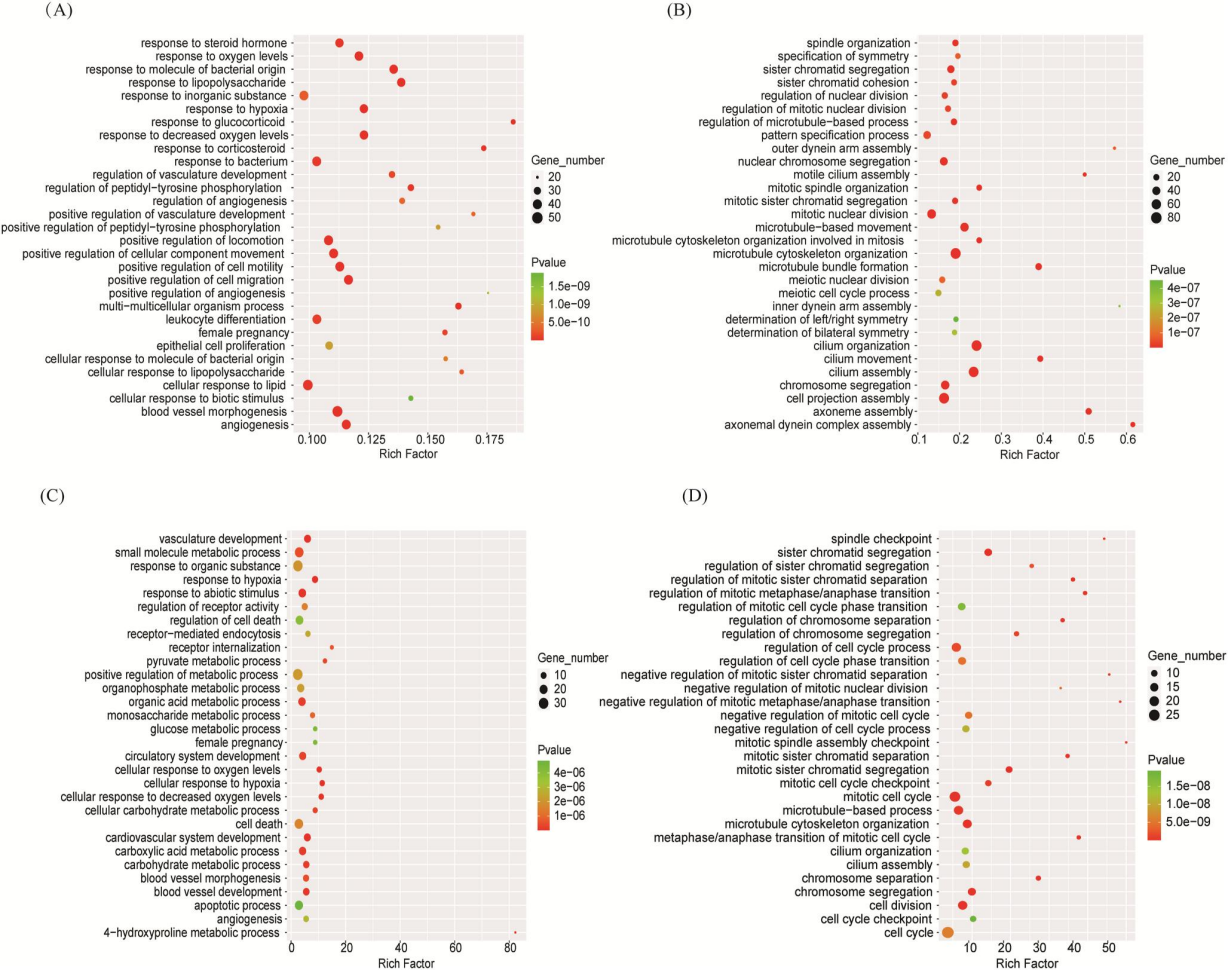
Top significantly enriched GO BP terms for DE‐mRNAs under hypoxia. The bubble charts show the top 30 GO BP terms enriched with significantly upregulated (A) and downregulated (B) genes in HNECs after 7 days of submerged culture (Submerged) compared to ALI control culture (ALI) (*n* = 5) and the top 30 GO BP terms enriched with significantly upregulated (C) and downregulated (D) genes after 7 days of 100 μM CoCl2 treatment (CoCl2) compared to untreated control (Untreated) (*n* = 5). “Gene number” indicates the number of DE‐mRNAs in a term. “Rich Factor” indicates the ratio of the number of DE‐mRNAs to the number of all mRNAs in a term

The DE‐mRNAs in the submerged culture and CoCl2‐treated groups were subjected to GO term enrichment analysis, and the GO BP terms enriched with upregulated or downregulated DE‐mRNAs are shown in Figure 2. Compared with the ALI control cells, the GO BP terms enriched with upregulated genes in the HNECs in the submerged culture were mainly associated with the hypoxia response (i.e., response to oxygen levels, response to decreased oxygen levels, and response to hypoxia), blood vessel morphogenesis, angiogenesis, the response to lipopolysaccharides, and the response to molecules of bacterial origin (Figure 2A), whereas the top GO BP terms enriched with downregulated genes included cilium organization, cilium assembly, cilium movement, microtubule cytoskeleton organization, and cell projection assembly (Figure 2B). In CoCl2‐treated cells, the GO BP terms most significantly enriched with the upregulated genes included response to hypoxia, vasculature development, cellular response to hypoxia, cellular response to decreased oxygen levels, 4‐hydroxyproline metabolic process, cellular response to oxygen levels, etc (Figure 2C), while the GO BP terms enriched with the downregulated genes were closely related to mitotic sister chromatid segregation, sister chromatid segregation, microtubule cytoskeleton organization, and cell division, as well as cilium assembly and cilium organization (Figure 2D). The upregulated gene‐enriched BP terms common to both hypoxia treatments included response to hypoxia, blood vessel morphogenesis, angiogenesis, and female pregnancy, whereas the downregulated gene‐enriched BP terms common to both hypoxia treatments were cilium organization, cilium assembly, microtubule cytoskeleton organization, chromosome segregation, sister chromatid segregation, and mitotic sister chromatid segregation.

**FIGURE 3:**
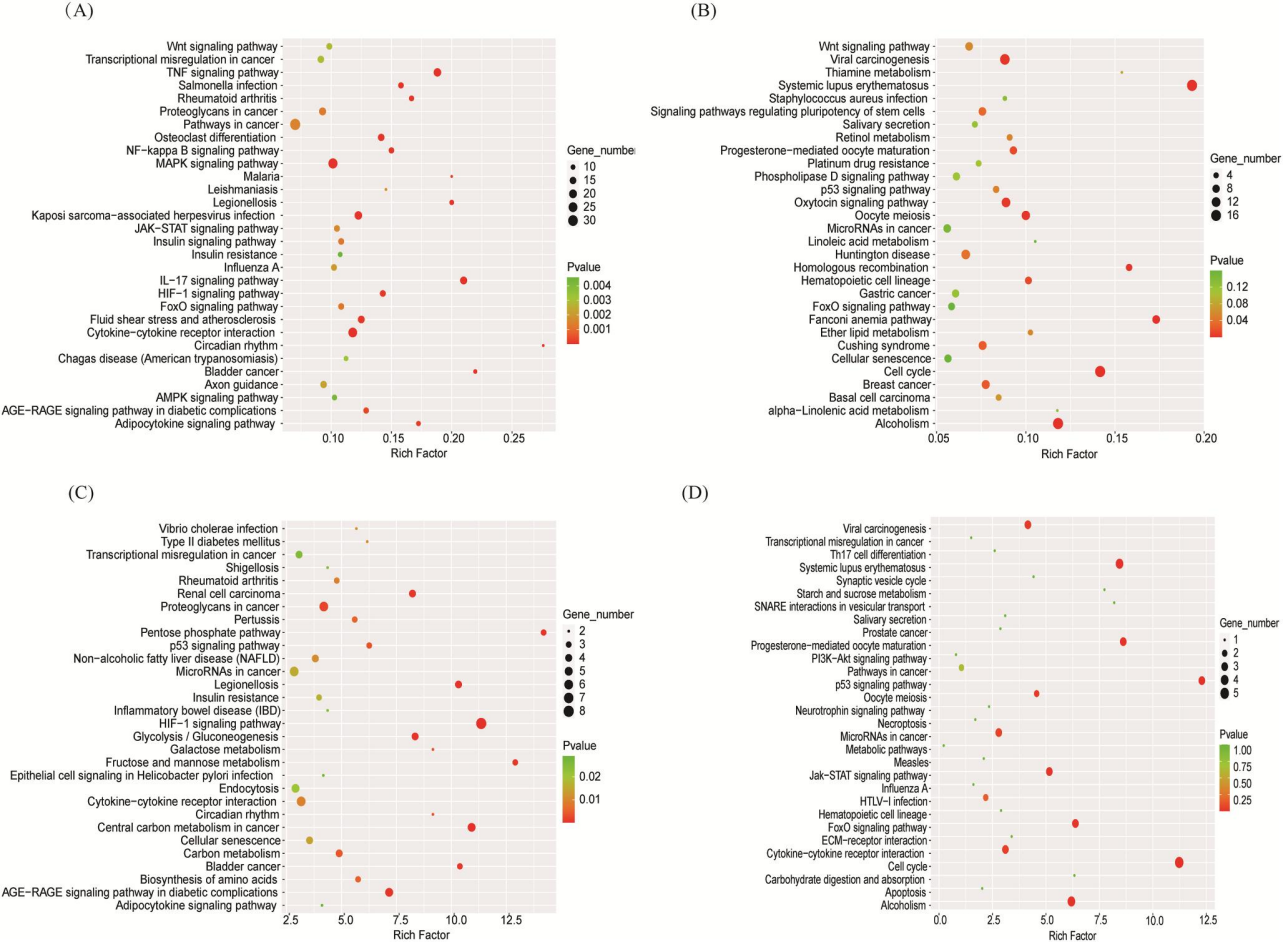
Top KEGG pathways significantly enriched with DE‐mRNAs under hypoxia. The bubble charts show the top 30 KEGG pathways enriched with significantly upregulated (A) and downregulated (B) genes in HNECs after 7 days of submerged culture (Submerged) compared to ALI control culture (ALI) (*n* = 5) and the top 30 KEGG pathways enriched with significantly upregulated (C) and downregulated (D) genes after 7 days of 100 µM CoCl2 treatment (CoCl2) compared to untreated control (Untreated) (*n* = 5). “Gene number” indicates the number of DE‐mRNAs in a pathway. “Rich Factor” indicates the ratio of the number of DE‐mRNAs to the number of all mRNAs in a pathway

A KEGG pathway enrichment analysis was performed to identify pathways enriched with DE‐mRNAs in the submerged culture versus ALI control groups and in the CoCl2 versus untreated control groups. As shown in Figure 3A,B, the KEGG pathways enriched with obviously upregulated genes in the submerged culture versus the ALI control group included the TNF signaling pathway, the IL‐17 signaling pathway, cytokine–cytokine receptor interactions, the MAPK signaling pathway, and the HIF‐1 signaling pathway, whereas the KEGG pathways enriched with downregulated genes included systemic lupus erythematosus, cell cycle, and alcoholism pathways, among others. The KEGG pathways enriched with upregulated genes between the CoCl2 and untreated control groups included the HIF‐1 signaling pathway, central carbon metabolism in cancer, legionellosis, and the pentose phosphate pathway, and those enriched with downregulated genes included cell cycle, the p53 signaling pathway, and systemic lupus erythematosus, among others (Figure 3C,D). In addition, several enriched pathways were shared by both treatment groups; for example, HIF‐1 signaling pathway, cytokine–cytokine receptor interaction, and adipocytokine signaling pathway were shared pathways enriched with upregulated genes, while systemic lupus erythematosus, cell cycle, and p53 signaling pathway were shared pathways enriched with downregulated genes.

### 3.3 Expression of cilia‐related genes

**FIGURE 4:**
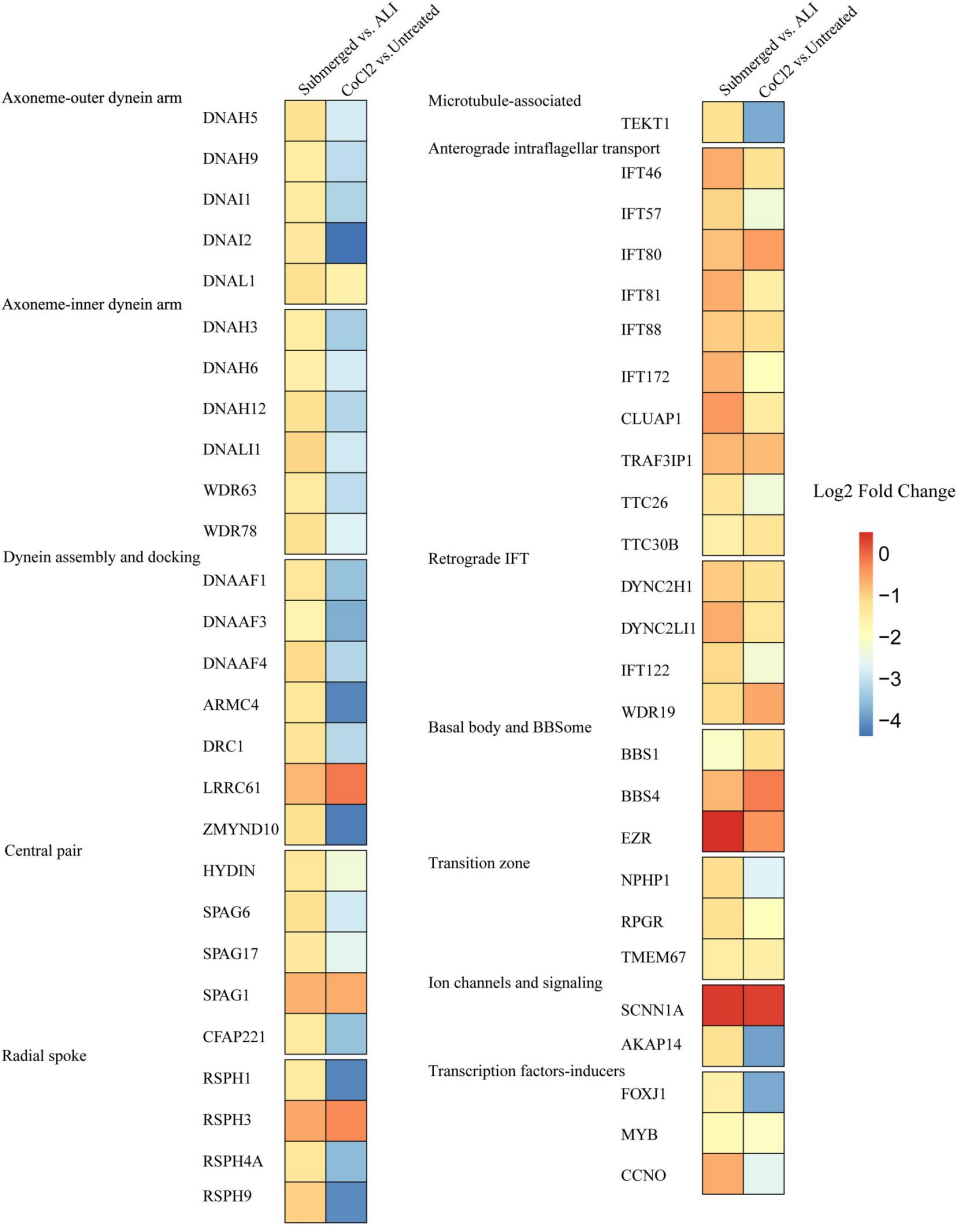
Changes in the expression of cilia‐related genes under hypoxia. The color coding in the first column of the heatmaps indicates the changes in the expression of cilia‐related genes as assessed by their log2 fold change expression values after 7 days of submerged culture (Submerged) compared to ALI control (ALI) culture (*n* = 5). The color coding in the second column of heatmaps indicates the changes in the expression of cilia‐related genes as assessed by their log2 fold change expression values after 7 days of 100 µM CoCl2 treatment (CoCl2) compared to untreated control (Untreated) (*n* = 5)

The above RNA‐seq data suggested alterations in the mRNA expression of cilia‐related genes; therefore, we further analyzed the expression of several genes related to ciliogenesis and ciliary function under these two hypoxic conditions. Numerous genes related to cilia were downregulated in the HNECs under both the submerged culture and CoCl2 treatment conditions, compared to the ALI control and untreated control, respectively; for example, genes encoding components of the axonemal outer dynein arm (DNAH5, DNAH9, DNAI1, DNAI2, and DNAL1) or inner dynein arm (DNAH3, DNAH6, WDR63, and WDR78), genes involved in dynein assembly and docking (DNAAF1, DNAAF3, ARMC4, and DRC1), genes related to the central pair (HYDIN, SPAG17, and CFAP221) or radial spoke (RSPH1 and RSPH4A), and, importantly, key transcription factors involved in ciliogenesis (FOXJ1 and MYB; Figure 4).

### 3.4 Validation of cilia‐related gene expression by RT–PCR

**FIGURE 5:**
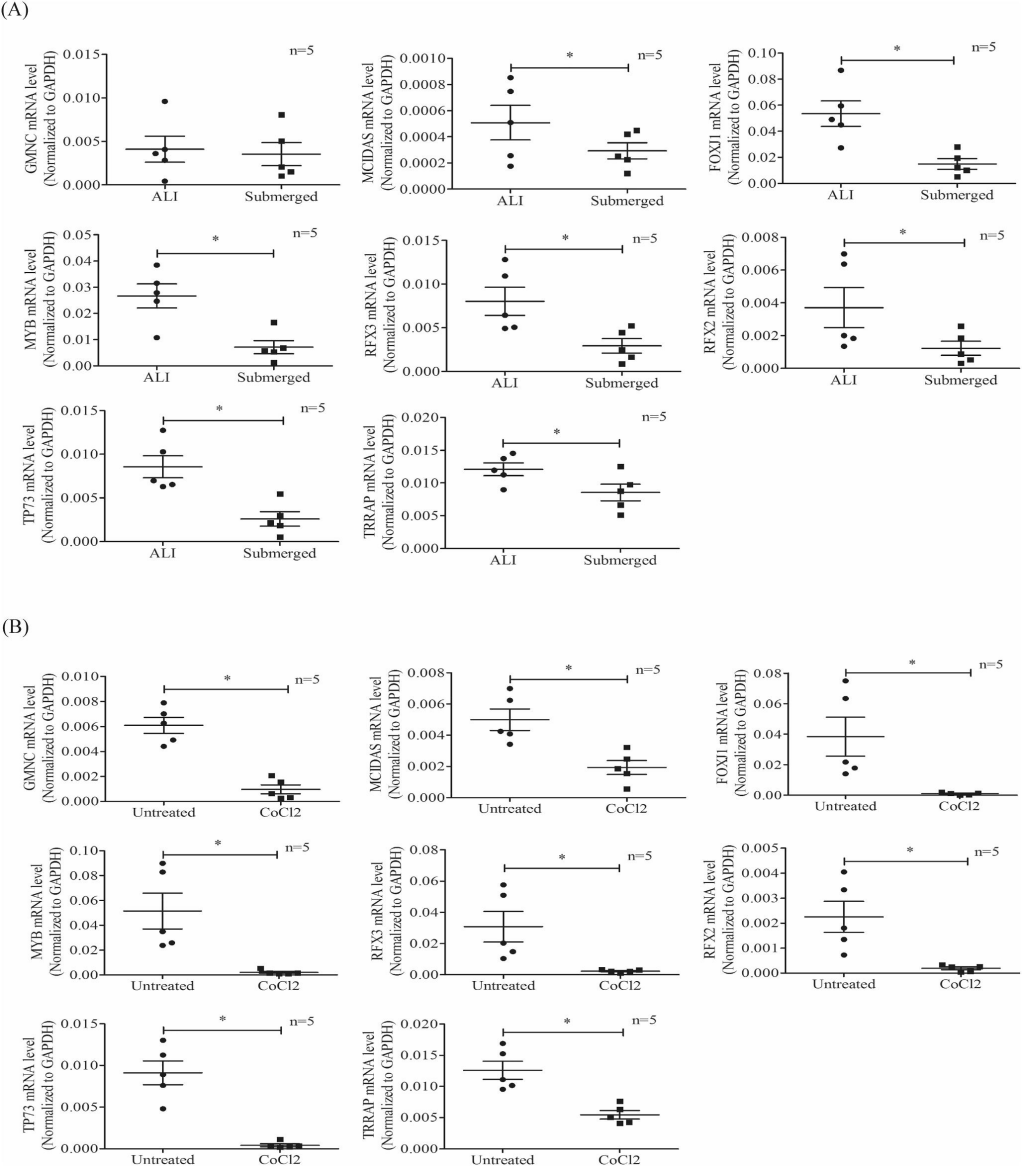
Changes in the mRNA expression levels of cilia‐related genes under hypoxia. (A) Changes of GMNC, MCIDAS, FOXJ1, MYB, RFX3, RFX2, TP73, and TRRAP mRNA expression levels as analyzed by RT–PCR after 7 days of submerged culture (Submerged) compared to ALI control (ALI) culture (*n* = 5). (B) Changes of GMNC, MCIDAS, FOXJ1, MYB, RFX3, RFX2, TP73, and TRRAP mRNA expression levels as analyzed by RT–PCR after 7 days of 100 µM CoCl2 treatment (CoCl2) compared to untreated control (Untreated) (*n* = 5). *p < 0.05 between the two groups

After 7 days of submerged culture or 100 µM CoCl2 treatment, the mRNA levels of cilia‐related genes (GMNC, MCIDAS, FOXJ1, MYB, RFX3, RFX2, TP73, and TRRAP) in HNECs were assessed by using RT–PCR. The mRNA levels of MCIDAS, FOXJ1, MYB, RFX3, RFX2, TP73, and TRRAP were significantly decreased in HNECs in submerged culture compared with the ALI control culture (Figure 5A). Similarly, the expression levels of all of the 8 detected cilia genes were significantly decreased in CoCl2‐treated cells compared with untreated control HNECs (Figure 5B).

### 3.5 Hypoxia inhibits ciliated cell differentiation

**FIGURE 6:**
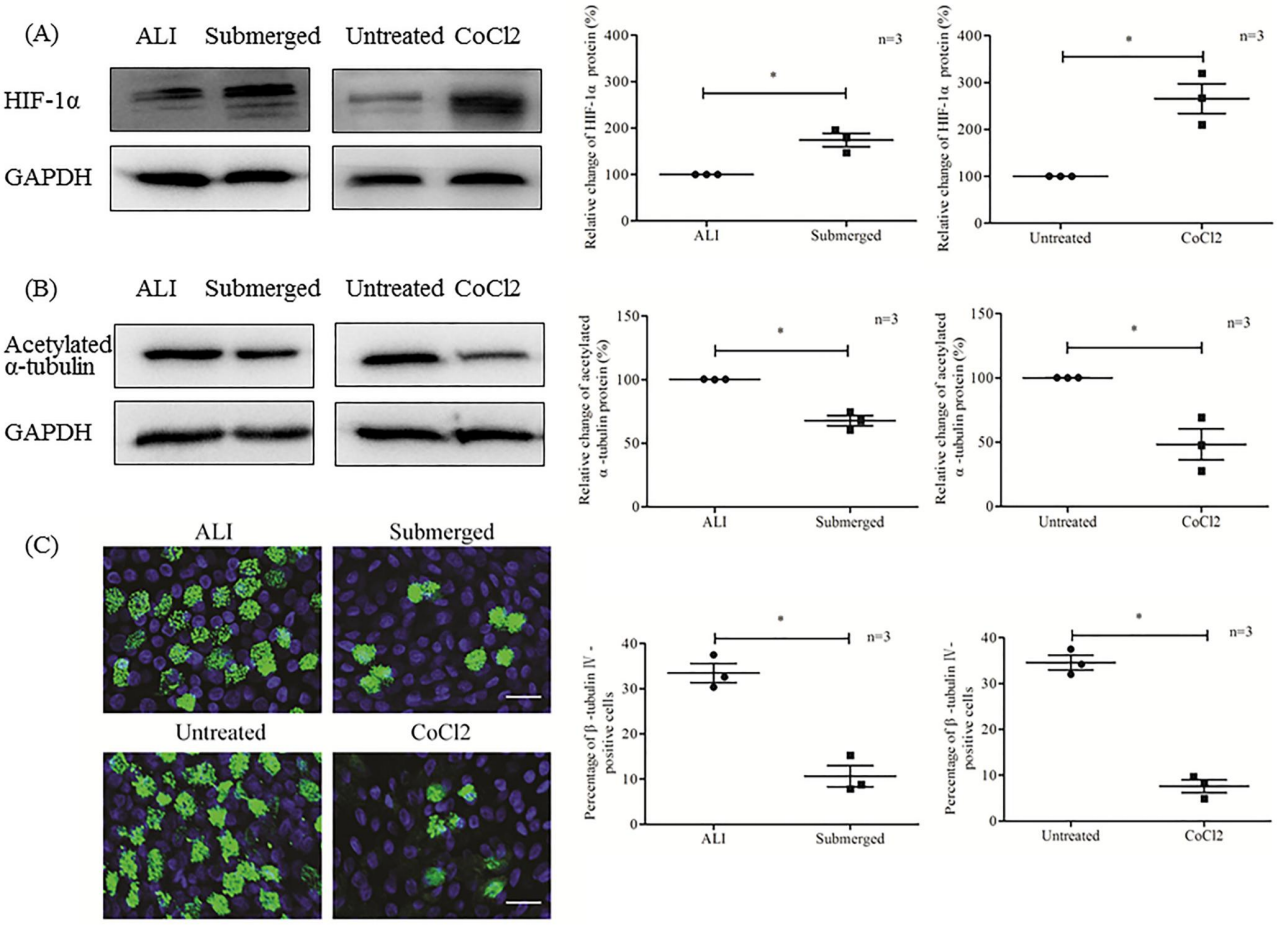
Changes in ciliated cell differentiation under hypoxia. (A) Changes of the protein level of HIF‐1α as measured by Western blotting after 4 h of submerged culture (Submerged) or 24 h of 100 µM CoCl2 treatment (CoCl2), compared to the ALI control (ALI) and untreated control (Untreated), respectively (*n* = 3). (B) Changes of the protein level of acetylated α‐tubulin (cilia marker) as measured by Western blotting after 7 days of submerged culture (Submerged) or 100 µM CoCl2 treatment (CoCl2), compared to the ALI control (ALI) and untreated control (Untreated), respectively (*n* = 3). (C) Changes in the differentiation of ciliated cells as shown by immunostaining for β‐tubulin IV (cilia marker) after 7 days of submerged culture (Submerged) or 100 μM CoCl2 treatment (CoCl2), compared to the ALI control (ALI) and untreated control (Untreated), respectively (*n* = 3). β‐tubulin IV (green) and nuclei (blue) are shown. Scale bar = 20 μm. *p < 0.05 between the two groups

To determine whether submerged culture or CoCl2 treatment induces hypoxia in HNECs, HNECs were maintained in submerged culture for 4 h or treated with 100 µM CoCl2 for 24 h. Subsequently, the protein level of HIF‐1α, which is a well‐known hypoxia marker, as well as the mRNA expression of several hypoxia‐response genes, were measured by using Western blotting and RT‐PCR, respectively. Both the submerged culture and CoCl2 treatment increased the expression of HIF‐1α protein (Figure 6A), as well as the mRNA levels of hypoxia‐response genes such as GLUT1, VEGF, BNIP3, and LDHA (Figure S2), thus indicating the establishment of a hypoxic environment.

Then, to investigate the effect of hypoxia on ciliated cell differentiation, HNECs were maintained in submerged culture or treated with CoCl2 for 7 days, and expression of the cilia markers acetylated‐α‐tubulin and β‐tubulin IV was detected by Western blotting and immunofluorescence staining, respectively. The protein level of acetylated α‐tubulin was obviously decreased under both treatment conditions (Figure 6B). Similarly, under both the submerged culture and CoCl2 treatment conditions, fewer β‐tubulin IV‐positive ciliated cells were observed than under the ALI control and the untreated control conditions, respectively (Figure 6C). These results further confirmed that hypoxia inhibited the differentiation of ciliated HNECs.

## 4 DISCUSSION

In this study, by using transcriptome sequencing, we analyzed the influence of hypoxia on the nasal epithelium function in primary cultured HNECs from patients with CRSwNP. The main finding was that the gene transcription program responsible for ciliogenesis and ciliary function was significantly impaired in the CRSwNP epithelium under hypoxic conditions. We have also validated the downregulation of cilia‐related genes and the inhibition of ciliated cell differentiation under hypoxia by using RT–PCR, Western blot, and immunofluorescence analyses.

The pathological mechanisms underlying CRSwNP remain unclear. Hypoxia due to occlusion of the sinus ostium is a major pathologic mechanism of CRSwNP. Shin et al reported that hypoxia‐induced epithelial‐to‐mesenchymal transition (EMT) is likely to contribute to nasal polyposis in CRS.^18^ Kim et al. demonstrated that hypoxia might upregulate MUC5AC via the HIF‐1α signaling pathway in HNECs and also found that hypoxia disrupted epithelial barrier function via production of VEGF and downregulation of junctional proteins.^9^, ^19^ Furthermore, hypoxia was reported to induce the normal nasal epithelium to produce more IL‐8, which is a major chemokine responsible for neutrophil recruitment and activation.^20^ Our study also detected the mRNA expression of IL‐8 under hypoxic conditions, and we found that both submerged culture and CoCl2 treatment induced an increased expression of IL‐8 in CRSwNP‐derived epithelial cells (Figure S2), thus implicating the role of hypoxia‐induced upregulation of IL‐8 in mediating neutrophil inflammatory responses in CRSwNP. Despite these findings, the mechanism of hypoxia in CRSwNP is not clearly understood. In the present study, by establishing two hypoxia models in vitro, we investigated the effects of chronic hypoxia on nasal epithelial function using RNA‐seq analysis combined with experimental validation. Both submerged and CoCl2 treatment significantly increased the expression of HIF‐1α and decreased the expression of cilia‐related genes and the differentiation of ciliated cells. These results might facilitate further exploration of hypoxia‐related mechanisms in the pathogenesis of CRSwNP.

Ciliated cells are the predominant cell type in the normal sinonasal epithelium, accounting for 50%–90% of respiratory epithelial cells. Each ciliated cell has more than 300 cilia to allow the clearance of mucus in cooperation with goblet cells, therefore maintaining a healthy airway environment. Substantial evidence indicates that impaired ciliogenesis or ciliary function is involved in the pathogenesis of CRSwNP.^21^ Li et al. reported that HNECs derived from CRSwNP tissue and differentiated in ALI culture exhibited marked defects in ciliary architecture,^22^ while Lai et al. showed that ciliogenesis was inhibited in patients with CRSwNP due to increased Cp110 expression.^23^ In a more recent study, Callejas‐Díaz et al. performed a transcriptome‐wide analysis during in vitro mucociliary differentiation of human adult basal stem cells (BSCs) from CRSwNP (compared to those isolated from the control mucosa), and their findings identified the transcriptional program responsible for multi‐ciliogenesis. Additionally, their results showed that cilia architecture/function was significantly impaired in differentiated CRSwNP cells (compared to control mucosa) at both the mRNA and miRNA levels.^8^ These studies provided strong evidence for cilia impairment in the CRSwNP epithelium; however, the mechanism underlying the ciliary defects in CRSwNP remains unclear. Our study demonstrated that hypoxia significantly inhibited the transcriptional program responsible for ciliogenesis and ciliated cell differentiation, thus suggesting that hypoxia may be a pathogenic mechanism underlying the ciliary defects in CRSwNP. Therefore, in the management of CRS, mechanical or pharmacological restoration of ventilation to alleviate hypoxia might be helpful for maintaining normal ciliary function.

A previous study by Gerovac et al. showed that the submerged culture creates a hypoxic environment for airway epithelial cells that is characterized by increased HIF‐1α and HIF‐2α expression.^17^ Although their study showed decreased ciliated cell differentiation in normal human bronchial epithelial (NHBE) cells, the comprehensive influence of submerged culture on epithelial cells remains unknown. Therefore, we first established an in vitro model of hypoxia in HNECs by submerged culture. After 7 days of submerged culture, the upregulated DE‐mRNAs were significantly enriched in BPs related to hypoxia or oxygen levels. In addition, the Western blot and RT‐PCR results showed an increased HIF‐1α protein level and the upregulation of hypoxia‐responsive genes, thus indicating the establishment of a hypoxic condition. Under this condition, BPs related to ciliary function, such as cilium organization, cilium assembly and cilium movement, were dramatically downregulated. Additionally, we found significantly decreased expression of many genes involved in ciliary architecture, such as genes related to the axoneme, dynein assembly and docking, or the central pair or radial spoke, as well as genes involved in ciliogenesis. The subsequent validated results of RT–PCR, Western blot analysis and immunofluorescence staining were consistent with the results of the bioinformatic analysis. Collectively, these results imply that hypoxia might alter ciliogenesis in HNECs.

Then, to further investigate the effects of hypoxia on ciliated cell differentiation, we treated cells with CoCl2, a well‐known chemical inducer of HIF‐1, to establish an in vitro chemical hypoxia model in HNECs and assessed the potential changes occurring under this condition. CoCl2 has been commonly used as a hypoxia‐mimicking agent both in vitro and in vivo to generate a hypoxia‐like environment by inducing the accumulation of HIF‐1α. It can also induce a series of genes that are involved in hypoxic signaling, such as VEGF, EPO, and GLUT1, which is similar to that observed in hypoxia.^24^ Our study showed that CoCl2 increased protein levels of HIF‐1α, as well as the mRNA expression of hypoxia‐response genes (Figure S2), thus confirming the establishment of a hypoxic model. RNA‐seq revealed that CoCl2 also dramatically enhanced the cellular processes related to hypoxia but inhibited the BPs related to cilium assembly and cilium organization and the expression of genes related to ciliary architecture and ciliogenesis. Similarly, the validation experiments showed that CoCl2 treatment significantly increased the expression of HIF‐1α and decreased the expression of a series of cilia‐related genes and the differentiation of ciliated cells. Taken together, our results provide strong evidence supporting the inhibition of ciliogenesis under hypoxia in HNECs. Further experiments are needed to elucidate the mechanisms underlying hypoxia‐mediated regulation of ciliary function in CRSwNP.

Notably, in addition to ciliogenesis‐related processes, other BPs were also common to the two hypoxia treatment conditions. For example, blood vessel morphogenesis and angiogenesis were upregulated in HNECs under both submerged culture and CoCl2 treatment conditions. Many studies have indicated that abnormal angiogenesis and vascular permeability are important for the formation of nasal polyps.^25^, ^26^ Additionally, hypoxia‐regulated angiogenesis has been reported to play an important role in various pathological conditions, such as solid tumors, vascular injury, and atherosclerotic lesion progression.^27^ These findings indicate that hypoxia might contribute to NP propagation by leading to dysregulated angiogenesis. We will continue to focus on the roles of epithelium‐derived angiogenic factors in the pathogenesis of nasal polyposis in follow‐up research.

Furthermore, the results obtained from the two datasets were considerably different due to the different mechanisms of action in the two hypoxia models. The submerged culture creates a hypoxic environment for epithelial cell growth; therefore, we applied this cell culture system to gain insight into the impact of hypoxia on epithelial function in vivo. CoCl2 treatment of in vitro‐cultured cells is a well‐established method for inducing changes similar to those seen under hypoxia.^28^ Our results showed that, in addition to enriching hypoxia‐ and cilia‐related BP terms, the submerged culture also notably enriched the BP terms related to inflammation and immunity, such as responses to lipopolysaccharide and responses to molecules of bacterial origin. Additionally, this culture depleted BP terms, such as cell projection assembly, axoneme assembly, and microtubule‐based movement. In addition, the upregulated DE‐mRNAs after CoCl2 treatment were closely linked to metabolic process terms such as 4‐hydroxyproline metabolic process, carboxylic acid metabolic process, carbohydrate metabolic process, and pyruvate metabolic process, while the downregulated DE‐mRNAs were notably enriched in the BP terms cell division, mitotic cell cycle, regulation of mitotic metaphase/anaphase transition, etc. These data may provide insights for further investigation of hypoxia‐mediated regulation of inflammation and immunity, metabolic processes, and other processes in the pathogenesis of CRSwNP.

The involvement of hypoxia‐induced immune responses in CRSwNP should be noteworthy, due to the fact that an increasing body of evidence indicated a distinct role for hypoxia in the regulation of immunity and inflammation. Hypoxia may lead to the activation of the HIF pathway in diverse cell types, such as cancer cells, epithelial cells, macrophages, and dendritic cells, and they are able to regulate innate and adaptive immunity. Hypoxia also plays an important role in the regulation of airway epithelial innate immune responses. Polke et al. reported that hypoxia suppresses innate immune functions of the airway epithelial cells, which contributes to microbial infection of mucosal lung surface in patients with chronic respiratory tract disease.^29^ Moreover, Sturrock et al. found that exposure of primary murine alveolar epithelial cells (AEC) to hypoxia resulted in significant suppression of key innate immune molecules, thus suggesting that local hypoxia may contribute to the susceptibility of the lung to infection through effects on reducing AEC expression of key innate immune molecules.^30^ Hypoxia was also demonstrated to be involved in the pathogenesis of CRSwNP through the regulation of Th17 immune responses of the nasal polyp epithelium.^31^ These data indicate that hypoxia‐induced dysregulation of the airway epithelial innate immune response may be associated with a compromised immunity and chronic inflammation of the airway, which highlight the essential work that is further needed to outline the role of tissue hypoxia in the regulation of immunity and inflammation in the pathophysiology of CRSwNP.

Our study had several limitations. First, we performed the experiments with only CRSwNP‐derived epithelial cells, but we did not include healthy control cells, which may decrease the relevance of the findings. Further studies for comparing the difference in responses to hypoxia between inflamed and non‐inflamed epithelial cells are necessary. Another limitation of this study was that the number of samples was small; thus, the sample sizes should be expanded for group comparisons of the effects of hypoxia on different phenotypes of CRSwNP. Thirdly, the molecular mechanisms underlying the influence of hypoxia on ciliary function remains to be elucidated. Lastly, in vitro experiments could not completely mimic in vivo conditions; therefore, these results need to be further verified in animal models in the future.

In summary, this study provides novel insight into the molecular basis of sinonasal ciliogenesis under hypoxic conditions, demonstrating that the gene transcription program responsible for ciliogenesis and ciliary function is significantly impaired under hypoxia, which might contribute to the disrupted mucociliary function in CRSwNP.

## AUTHOR CONTRIBUTIONS

Jian Jiao wrote the manuscript. Jian Jiao, Puqi Hu, Xiangdong Wang, and Luo Zhang designed the study and revised the manuscript. Puqi Hu, Mengyan Zhuang, and Ying Li performed the experiments. Jian Jiao, Puqi Hu and Chao Cai analyzed the data.

## CONFLICT OF INTEREST

The authors report no conflict of interest.

## Supporting information

Supplementary Material

## Data Availability

Data available on request from the authors.
