# Supplementary material for: Transcriptome sequencing reveals altered ciliogenesis under hypoxia in nasal epithelial cells from chronic rhinosinusitis with nasal polyps
Source: Clin Transl Allergy. 2022 Jun 8;12(6):e12168. doi: 10.1002/clt2.12168 (PMC9174880; doi:10.1002/clt2.12168)

**SUPPLYMENTARY TABLES**

Table S1. The demographics and clinical characteristics of the CRSwNP patients

|  | Submerged culture treatment group (N=5) | CoCl2  treatment group (N=5) |
| --- | --- | --- |
| Age (y) | 52 (28-68) | 42 (37-67) |
| Male, n (%) | 4 (80) | 4 (80) |
| Smoker, n (%) | 2 (40) | 1 (20) |
| AR, n (%) | 1 (20) | 1 (20) |
| Asthma, n (%) | 1 (20) | 1 (20) |
| Tissue Eos (number/HPF) | 22 (6-110) | 25 (2-76) |
| Blood Eos (%) | 1.5 (0.9-2.4) | 2.7 (0.3-3.8) |
| Previous surgery, n (%) | 2 (40) | 1 (20) |

Note: Data are expressed as numbers (%), or median (range).

Abbreviations: AR, allergic rhinitis; Eos, eosinophil; HPF, high power field.

Table S2. Primers used for real-time PCR

| Primers | Forward | Reverse |
| --- | --- | --- |
| GMNC | 5’-GCACCACAGGCACAGGAATC -3’ | 5’-CTTCGTGTAACCTGGCGAGT-3’ |
| MCIDAS | 5’-CAGCCCTCACCACCATAGAC -3’ | 5’-GGTGGGAATGGTTCTGCGA-3’ |
| FOXJ1 | 5’-GTGGGAGCAACTTCTTCCAGA-3’ | 5’-ATAAGTATGTGGTGCCTGGCT-3’ |
| MYB | 5’-GGGAACAGATGGGCAGAAATCG -3’ | 5’-GCTGGCTTTTGAAGACTCCTGC-3’ |
| RFX3 | 5’-CAGTCATGGGCGAGTTTGGT-3’ | 5’-ACAACAGTCGACCTTCAGGC-3’ |
| RFX2 | 5’-TGGGATTCGTCTGAAGCCG-3’ | 5’-GGAGACATCTATGTACTGCTGGT-3’ |
| TP73 | 5’-CGGGCCATGCCTGTTTACA-3’ | 5’-TGTCCTTCGTTGAAGTCCCTC-3’ |
| TRRAP | 5’-GTGGACCTGTCTGAAGTCGTCA-3’ | 5’-TCACTTCCTGGGCAGAATCCAC-3’ |
| IL8 | 5’-GAAGTTTTTGAAGAGGGCTGAGA-3’ | 5’-GGCACAGTGGAACAAGGACT-3’ |
| GLUT1 | 5’-CACTGTCGTGTCGCTGTTTG-3’ | 5’-CAAGTGTCTCGACAGGGCTT-3’ |
| VEGF | 5’-GCAGAATCATCACGAAGTGGT-3’ | 5’-ACCAACGTACACGCTCCAG-3’ |
| BNIP3 | 5’-TGGACGGAGTAGCTCCAAGA-3’ | 5’-CGCCTTCCAATATAGATCCCCAA-3’ |
| LDHA | 5’-TTCCGGATCTCATTGCCACG-3’ | 5’-CCACTCCATACAGGCACACT-3’ |
| GAPDH | 5’-GGAGCGAGATCCCTCCAAAAT-3’ | 5’-GGCTGTTGTCATACTTCTCATGG-3’ |

**SUPPLYMENTARY FIGURE LEGENDS**

Figure S1. Timeline scheme of the study design.


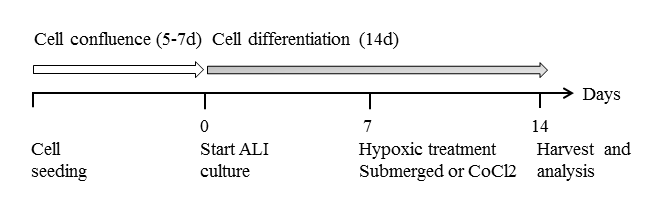


Figure S2. Changes in the mRNA expression levels of IL-8, GLUT1, VEGF, BNIP3, and LDHA under hypoxia. (A) Changes in IL-8, GLUT1, VEGF, BNIP3, and LDHA mRNA expression levels, as analyzed via RT–PCR after 7 days of submerged culture (Submerged) compared to ALI control (ALI) culture (n=5). (B) Changes in IL-8, GLUT1, VEGF, BNIP3, and LDHA mRNA expression levels, as analyzed via RT–PCR after 7 days of 100 μM CoCl2 treatment (CoCl2) compared to the untreated control (Untreated) (n=5). *P < 0.05 between the two groups.


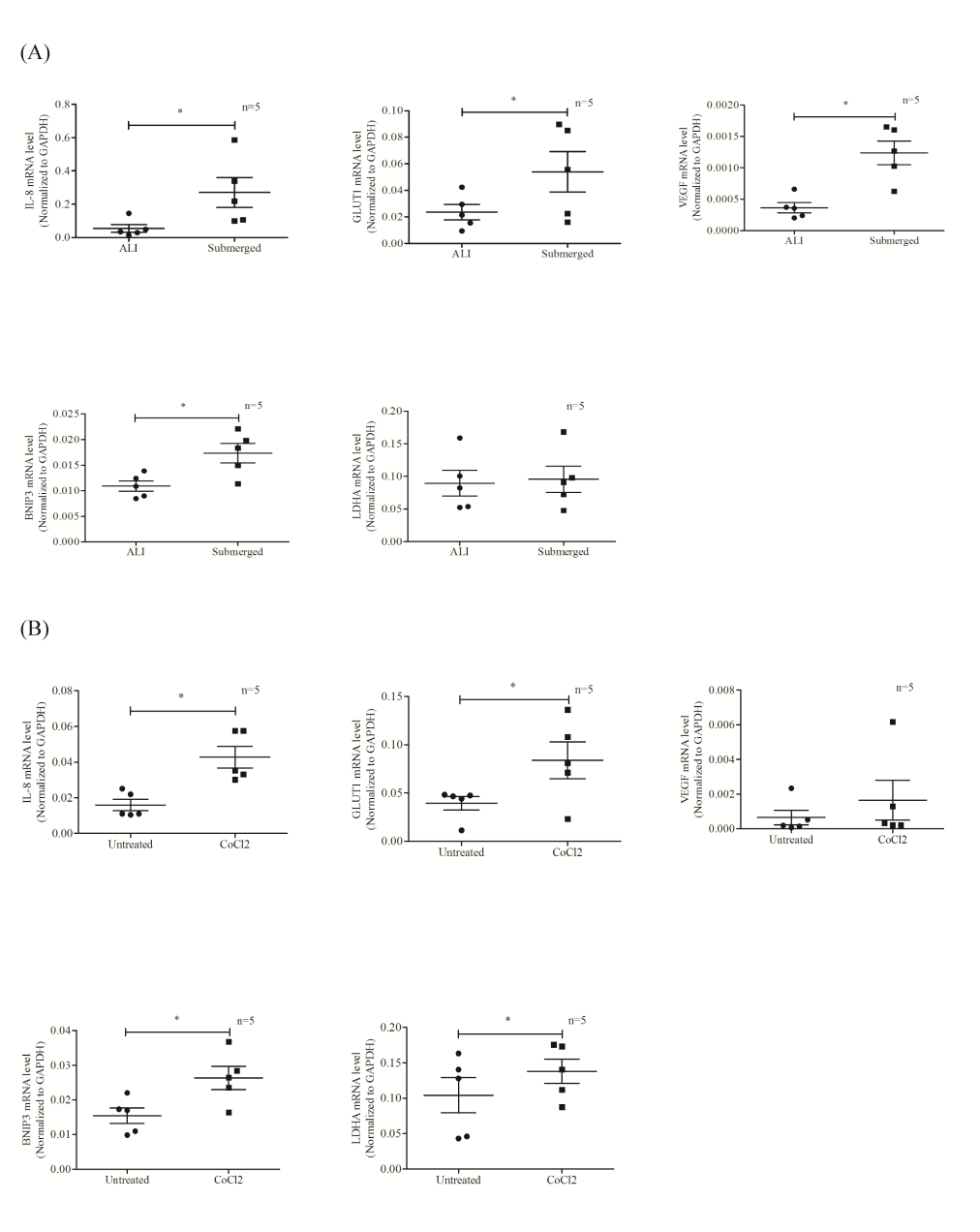

Supplement: Supplementary file 1 — Supplementary Material [file CLT2-12-e12168-s001.docx]
